# Editorial: Ketogenic Diet in Epilepsy and Associated Comorbidities: Clinical Efficacy and Mechanisms

**DOI:** 10.3389/fneur.2020.00066

**Published:** 2020-02-19

**Authors:** Tianfu Li, Jiong Qin, Xiang-Ping Chu

**Affiliations:** ^1^Department of Neurology, Beijing Institute for Brain Disorders, Beijing Key Laboratory of Epilepsy Research, Sanbo Brain Hospital, Capital Medical University, Beijing, China; ^2^Department of Pediatrics, Peking University People's Hospital, Beijing, China; ^3^Departments of Biomedical Sciences and Anesthesiology, School of Medicine University of Missouri-Kansas City, Kansas City, KS, United States

**Keywords:** epilepsy, comorbididites, ketogenic diet, epileptogenesis, mechanism

Epilepsy is a chronic disease of the brain with the characteristic of a long-suffering propensity to create epileptic seizures and the associated comorbidities including neurobiological, cognitive, psychological, and social consequences of this condition. According to the practical clinical definition of the International League Against Epilepsy, the diagnosis of epilepsy needs at least two unprovoked (or reflex) seizures occurring >24 h apart, one unprovoked (or reflex) seizure and a probability of further seizures similar to the general recurrence risk after two unprovoked seizures occurring over the next 10 years, or a diagnosis of an epilepsy syndrome (1). Currently, up to 30 percent of patients with epilepsy are pharmacoresistant (2, 3), and apart from those who are candidates for resective surgery, most will continue to suffer from refractory seizures and associated cognitive and psychiatric comorbidities. Currently, no conventional antiepileptic drugs have been validated to preclude epileptogenesis or epilepsy-related comorbidities. Therefore, to develop the novel drugs is urgently needed. Recently, extensive clinical and experimental evidence demonstrates the ketogenic diet (KD) with the promising benefit to ameliorate both the epileptic seizures and associated comorbidities in patients with pharmacoresistant epilepsy (Chen et al.).

As editors of this special edition on ketogenic diet in epilepsy and associated comorbidities: clinical efficacy and mechanisms, we are pleased to manifest the collection of papers featured in this Research Topic. The final collection of this Research Topic comprises two original articles and four original research reviews.

The original clinical research article by Tian et al. demonstrated that KD therapy provided good clinical efficacy for seizure control, reduction of the frequency of interictal epileptic discharges, as well as improvement of the cognitive function and language ability in patients with Dravet Syndrome. The experimental research article by Zhu et al. revealed that ketone bodies (including acetoacetic acid, β-hydroxybutyric acid, and acetone) in KD undermined the currents of acid-sensing ion channels in rat hippocampal neurons, which indicated that inhibition the open of acid-sensing ion might be the underlying antiepileptic mechanism of KD therapy for the drug-resistant epilepsy.

In the review papers, the review from Ren et al. addressed the characteristics of a Model of Sudden Unexpected Death in Epilepsy (SUDEP) in an animal model of SUDEP in Kcna1-null mouse. The review evaluated that KD therapy could prolong longevity and inhibit seizure progression in Kcna1-Null mouse. In addition, the underlying mechanisms of action of KD therapy in control seizure and SUDEP are demonstrated as well. Liu et al. performed a systematic review and meta-analysis to explore the relation of spasm remission between the 3 and 6 months KD treatment for intractable childhood epilepsy. The data indicated that a period of 3 months of KD as a predictor of 6 months duration and 3 months might be taken as a course of treatment duration in terms of spasm remission.

The review from Yang et al. evaluated the neuroprotective effects of KD in neurodegenerative diseases such as epilepsy, Parkinson's disease, Alzheimer's disease, and traumatic brain injury. In addition, anti-oxidative stress, maintaining energy supply, modulating the activity of deacetylation and inflammatory responses were indicated as the potential mechanisms of KD therapy in the treatment of the diseases mentioned above. Li et al. assessed the KD efficacy in ameliorating both seizures and comorbidities associated with epilepsy, such as cognitive/psychiatric concerns for the patients with refractory epilepsy. The potential mechanisms of action of the KD therapies focused on the adenosine system in the prevention of epileptogenesis and comorbidities associated with epilepsy are well-illustrated in the review.

## Author Contributions

TL wrote this editorial. JQ and X-PC commented on the amendments.

### Conflict of Interest

The authors declare that the research was conducted in the absence of any commercial or financial relationships that could be construed as a potential conflict of interest.
